# Exosome Analysis in Prostate Cancer: How They Can Improve Biomarkers’ Performance

**DOI:** 10.3390/cimb45070384

**Published:** 2023-07-21

**Authors:** Stefano Salciccia, Marco Frisenda, Giulio Bevilacqua, Luca Gobbi, Bruno Bucca, Martina Moriconi, Pietro Viscuso, Alessandro Gentilucci, Gianna Mariotti, Susanna Cattarino, Flavio Forte, Stefano Fais, Mariantonia Logozzi, Beatrice Sciarra, Alessandro Sciarra

**Affiliations:** 1 Department Materno Infantile Scienze Urologiche, University Sapienza, Viale Policlinico 155, 00161 Rome, Italy; stefano.salciccia@uniroma1.it (S.S.); marco.frisenda@uniroma1.it (M.F.); giulio.bevilacqua@uniroma1.it (G.B.); luca.gobbi@uniroma1.it (L.G.); bruno.bucca@uniroma1.it (B.B.); martina.moriconi@uniroma1.it (M.M.); pietro.viscuso@uniroma1.it (P.V.); alegenti@yahoo.com (A.G.); gianna.mariotti@gmail.com (G.M.); susycat84@hotmail.it (S.C.); 2 Figliesancamillo Hospital, 00198 Rome, Italy; flavio.forte@figliesancamillo.it; 3 Istituto Superiore di Sanita, Viale Regina Elena, 00161 Rome, Italy; 4 Department of Chemistry, University Sapienza, Viale Universita, 00161 Rome, Italy

**Keywords:** exosome, prostate cancer, biomarker

## Abstract

Exosomes are extracellular nanovesicles (EV), that is, carriers of different biomolecules such as lipids, proteins, nucleic acids. Their composition and the fact that their release dramatically increases in cases of tumorigenesis open up different scenarios on their possible application to research into new biomarkers. The first purpose of the present review was to specifically analyze and compare different methodologies available for the use of exosomes in prostate cancer (PC). The most widely applied methodologies include ultracentrifugation techniques, size-based techniques, immunoaffinity capture-based techniques (mainly ELISA), and precipitation. To optimize the acquisition of exosomes from the reference sample, more techniques can be applied in sequence for a single extraction, thereby determining an increase in labor time and costs. The second purpose was to describe clinical results obtained with the analysis of PSA-expressing exosomes in PC; this provides an incredibly accurate method of discriminating between healthy patients and those with prostate disease. Specifically, the IC-ELISA alone method achieved 98.57% sensitivity and 80.28% specificity in discriminating prostate cancer (PC) from benign prostatic hyperplasia (BPH). An immunocapture-based ELISA assay was performed to quantify and characterize carbonic anhydrase (CA) IX expression in exosomes. The results revealed that CA IX positive exosomes were 25-fold higher in plasma samples from PC patients than in those from healthy controls. The analysis of PC-linked exosomes represents a promising diagnostic model that can effectively distinguish patients with PC from those with non-malignant prostatic disease. However, the use of exosome analysis in clinical practice is currently limited by several issues, including a lack of standardization in the analytical process and high costs, which are still too high for large-scale use.

## 1. Introduction

Prostate tumors are the most commonly diagnosed tumor in the male population, with an estimated 1.4 million new diagnoses and 300,000 related deaths worldwide in 2020. Nowadays, the gold standard for its diagnosis is prostate biopsy, which is usually performed after clinical suspicion, initially based on prostate-specific antigen (PSA) value and digital rectal examination (DRE). However, the reduced sensitivity of the DRE and the low specificity of the PSA have led to the research into new methods of early and non-invasive diagnosis for prostate cancer (PC) [1]

Exosomes are extracellular nanovesicles (30–100 nm), that is, carriers of different biomolecules such as lipids, proteins, nucleic acids, which are secreted from all mammalian cells under normal and pathological conditions. Their role as extracellular messengers is similar to that of a message in a bottle traveling in our body [2]. Their composition and the fact that their release dramatically increases in case of tumorigenesis open up different scenarios on their possible application to research into new biomarkers. The ability of exosomes to transmit their content of lipids, proteins, DNA, mRNA, and other metabolites into the target cells confers a crucial role in the intercellular communication and modulation of physiological or pathological processes, including tumor progression. For these reasons, exosomes can be considered an ideal source of new and more specific tumor biomarkers. From an analytical point of view, exosomes are not easy to study mainly due to their size. Tumor-derived exosomes can be detected in our fluids and can provide diagnostic or prognostic information [3]. Pre-clinical evidence has shown that increased levels of plasmatic exosomes are directly related to the presence of a neoplastic mass, and that surgical treatment of the primary tumor led to a significant reduction in their plasmatic levels [3]. Our group finalized different research on exosome analysis in patients with prostate cancer, obtaining significant results. The purpose of the present editorial is to summarize the methodology used, the results obtained, and the limits found in our personal experience. We will try to underline how research on exosome could allow us to obtain markers with higher specificity for neoplastic disease in the prostate, but the complexity of the analytical methodology required is an obstacle to overcome. In particular, and differing from previous reviews, (1) the methodology to be used for exosome analysis in PC is specifically described; (2) we specifically analyzed how exosome analysis can improve the specificity and sensitivity of the major marker used in PC diagnosis, PSA (PSA expressing exosomes); and (3) we specifically analyzed the relationship between exosomes and carbonic anhydrase expression in PC.

## 2. Biomarkers in Prostate Cancer

A biomarker is defined as an indicator of the normal or pathological biological process of a cell or tissue that is able to differentiate between the two conditions [4]. The main characteristics of a biomarker are its specificity and sensitivity for the pathologic condition, and its usefulness with regard to early diagnosis, prognosis, and therapeutic indication.

Tumor biomarkers for PC can be obtained from serum, urine, and tissue samples. To date, urine samples are the simplest to obtain from the patient and the most abundant, containing important amounts of proteins, cellular DNA, and RNA, as well as cellular metabolites and exosomes, which can represent the basis to obtain a reliable biomarker for PC [5]. Urine is not associated with proteolytic processes as it does not cross tissue barriers, and this may represent a relevant advantage in the isolation process when compared to blood [6].

### 2.1. Serum Biomarkers

Prostate-specific antigen (PSA) is a glycoprotein produced mainly by the prostate glandular tissue, and it is secreted in the seminal fluid. It is an enzyme belonging to the hydrolase class, structurally similar to kallikrein (also called kallikrein 3). PSA is not a cancer-specific marker but an organ-specific marker, and its levels can be affected by non-neoplastic conditions such as prostatitis and benign prostatic hyperplasia (BPH) [7]. PSA is considered a continuous parameter, so that elevated PSA levels indicate a higher likelihood of PC and a higher risk of high-grade malignancy. In evaluating PSA for the early diagnosis of PC, the area under the curve (AUC) was found to be 0.68, while the AUC of PSA in discriminating between a clinically significant and non-significant PC is 0.78 [8].

The 4K score (OPKO Lab, Nashville, TN, USA) considers the serum levels of four kallikrein proteins, including PSA, free PSA, intact PSA and human kallikrein 2 (hK2), combining them with data from the digital rectal examination, age, and previous prostate biopsy, through an algorithm that therefore allows us to calculate the risk of high-grade PC [9]. It is recommended for patients who have already had a previous prostate biopsy, and the AUC for the initial diagnosis of PC was between 0.69 and 0.83 [9].

### 2.2. Urine Biomarkers

The Select MDx test (MDx Health, Irvine, CA, USA) is a reverse-transcriptase polymerase chain reaction (RT-PCR) that evaluates the *HOXC6* and *DLX1* mRNA gene levels in urine samples after prostate massage, in correlation with the risk of PC and high-grade PC. The AUC for the early diagnosis of high-grade PC ranged between 0.86 and 0.90 when combined with clinical parameters [10].

A biomarker measured in urine after prostate massage is the Fusion trans-membrane serine protease 2 (TMPRSS2) plus v-ets erythroblastosis virus E26 oncogene homolog (ERG) fusion gene, evaluated by reverse transcription polymerase chain reaction (RT-PCR) and fluorescence in situ hybridization (FISH). Both are located on chromosome 21; TMPRSS2 is a prostate-specific and androgen-responsive gene expressing a serine protease protein, operating in prostate carcinogenesis and based on gene fusion with ETS transcription factors (ERG and ETV1) [11]. ERG is an oncogene that encodes for a transcription factor, a member of the erythroblast transformation-specific family, which is a key regulator of cell proliferation, differentiation, angiogenesis, inflammation, and apoptosis [11]. 

The Michigan Prostate Score (MiPS) (University of Michigan, MLabs) is a predictive model that incorporates serum PSA, urinary PCA3 mRNA, and urinary TMPRSS2-ERG. The sample is collected immediately before prostate biopsy and requires 20–30 mL of urine, obtained after three prostate massages. The AUC in PC risk prediction was 0.75 and 0.77 for high-risk PC [12].

The ExoDx Prostate IntelliScore (EPI) urine exosome test (Exosome Diagnostics, Inc., Waltham, MA, USA) analyzes exosomes derived from normalized PCA3 and ERG RNA in urine specimens without the need for a prior prostate massage [13]. When the EPI is combined with other clinical parameters, it reduces the number of useless biopsies by 27% and it provides additional predictive accuracy in detecting clinically significant PC, with an AUC of 0.80 [14].

### 2.3. Tissue Biomarkers

The ConfirmMDx test (MDxHealth, Inc., Irvine, CA, USA) determines the level of methylation of the promoter regions of three genes involved in cell regulation (APC–adenomatous polyposis coli, RASSF1–ras association (RalGDS/AF-6) domain family member 1 and GSTP1–glutathiones-transferase PI1) in prostate tissues. DNA methylation occurs early during the oncogenic cycle and in the presence of cancer lesions; the perilesional tissue undergoes epigenetic modifications, which are detected by the test [15]. The test is performed using a multiplexed quantitative DNA methylation-specific polymerase chain reaction assay for the three genes considered [16]. The AUC for the prediction of clinically significant PC reached 0.76 [16].

The Prolaris score (Myriad Genetics, Inc., Salt Lake City, UT, USA) analyzes 46 genes (31 genes involved in cellular biology, and 15 housekeeping genes) via RT-PCR in order to predict the risk of 10-year disease-specific mortality and distant metastasis in PC cases. It is performed on both formalin-fixed paraffin-embedded (FFPE) needle biopsy and FFPE radical prostatectomy tissue samples. The test showed an AUC of 0.878 with regard to the prediction of PC, when combined with clinical parameters [17]. 

### 2.4. Exosome-Based Biomarkers

EV include apoptotic bodies, exosomes, and microvesicles. To date, many studies are focusing on the development of exosome-based diagnostic tests. Exosomes are small extracellular vesicles originating as cytoplasmic invaginations of the endosomes, which are produced by a wide range of normal and neoplastic cells; they are spilled over into body fluids, so they are easily detectable in serum, urine, and semen samples [18]. They are subsequently expelled after a mechanism of multivesicular body fusion with the plasma membrane [19], showing specific markers derived from endosomes, such as tetraspanins, heat shock proteins, and compounds of the Rab family [19]. It is possible to find nucleic acid derivatives (DNA, mRNA, microRNA) in the exosomes, and their function is probably a mediation of cell-to-cell communication or microenvironment modulation.

A characteristic phenotype of cancer cells is the development of an acidic microenvironment. This trait, closely linked to a hyperfunction of the proton pumps, guarantees increased survival for cancer cells, since normal cells do not survive in an acidic environment. The acidity of the microenvironment significantly increases the release of exosomes from cancer cells. Consequently, in the oncogenic process, there is a significant increase in the spill-over of exosomes from neoplastic cells, regardless of the histotype of the tumor [20]. These concepts led us to postulate that exosomes may be considered tumor biomarkers, with potential use in the screening, diagnosis, and prognosis of the disease.

## 3. How to Measure Exosome for Prostate Cancer Characterization

A crucial factor in the study of exosomes is their correct isolation. The most widely applied methodologies include ultracentrifugation techniques, size-based techniques, immunoaffinity capture-based techniques (mainly ELISA), and precipitation. To optimize the acquisition of exosomes from the reference sample, more techniques can be applied in sequence for a single extraction, thereby determining an increase in labor time and costs. 

The analysis of the obtained EV can be performed using physical analysis techniques (mainly nanoparticle tracking analysis (NTA), and flow cytometry) and compositional analysis techniques (mainly Western blotting) (Table 1) [21].

Nanoparticle tracking analysis (NTA), immune capture-based technologies, and nanoscale flowcytometry (NFC) represent valid technologies for analyzing extracellular vesicles and exosomes in samples with quantitative and qualitative information [2,3,22,23].

Moreover, EV at the plasma level can be characterized and quantified using an immunocapture-based ELISA (IC-ELISA) test [2,22]. For example, the association of these techniques well described the role of PSA-expressing exosomes, and they showed that acidic conditions stimulate exosome release from tumor cells [22,23].

Comparing the different techniques for detecting plasmatic EV in human fluids, several factors have made IC-ELISA a very interesting tool:

(a) it is non-invasive;
(b) it is rapid, specific, and quantitative;
(c) it requires a small quantity of sample, and is reproducible; and
(d) it is affordable, incurring reasonable costs in laboratories.

Preparation of plasma to obtain EV requires EDTA-treated blood, extraction of plasma, collection at −80 °C, and centrifugation in order to eliminate cell debris, organelles, and microvesicles. In the last step, plasma samples are centrifuged for 1 h 30 min at 110,000× *g* using a Fiberlite™ F50L-24 × 1.5 Fixed-Angle Rotor, K-factor: 33, from the Sorvall WX Ultracentrifuge Series, to obtain the exosomal pellets, which are then washed in PBS and resuspended in the appropriate buffer for subsequent analyses.

Nanoparticle Tracking Analysis (NTA) can be used for the measurement of the size distribution and concentration of extracellular vesicle samples in liquids [22]. It allows us to capture, detect, characterize, and quantify extracellular vesicles in both human body fluids and cell culture supernatants. It is based on the use of two antibodies, one directed against a typical exosomal housekeeping protein, and the second against either another exosomal housekeeping protein or a potential disease marker. The first antibody is used for the capture of exosomes, and the second for the quantification and characterization of the captured vesicles. In fact, with this method, it is possible both to characterize and count exosomes, and to detect the presence of disease, including the tumor and biomarkers. This requires preliminary purification of the extracellular vesicles of the clinical sample, and the most highly recommended method for this is repeated rounds of ultracentrifugation. This methodological approach allows us not to exclude the extracellular vesicle subpopulation from the separation procedure, and to analyze a full range of them from both a qualitative and quantitative point of view. 

Following laser beam illumination, the light scattering allowed us to visualize, record, and track the particles with a CCD or CMOS camera.

NTA 3.0 software can first identify and then track each particle on a frame-by-frame basis. NTA is based on the phenomenon of the random movement (diffusion) of small particles when they are dispersed in a liquid, allowing direct and precise measurement of the concentration and size of the particles. The Brownian motion of each particle is tracked using the Stokes–Einstein equation: D = kT/6πηr, where D is the diffusion coefficient, kT/6πηr is the frictional coefficient of the particle, and T is the absolute temperature.

The Immnocapture IC-ELISA test demonstrates clinical potentiality for PSA exosome evaluation. An antibody specific for a typical exosome antigen (CD81) is used to identify exosomes within the pool of extracellular vesicles, and an antibody for PSA is used for the detection of plasmatic exosomes expressing PSA. Nanovesicles purified from plasma are quantified using a Bradford assay, and then suspended in a final volume of 50 μL and incubated overnight at 37 °C. After three washes with PBS, a mouse anti-PSA HRP-conjugated is added, the reaction is developed with Blue POD for 15 min and blocked with 4N H2SO4 stop solution. Optical densities are recorded at 450 nm, and a PSA calibration curve allows us to convert the optical densities of each sample into micrograms of Exo-PSA [22,23].

In the Flow Cytometry Analysis of Exosomes, EV purified from plasma are diluted in PBS in a final volume of 50 μL. Anti-human CD81 allophycocyanin (APC)-conjugated, anti-human PSA fluorescein (FITC)-conjugated, and IgG1 FITC are added to the exosome preparation at optimal pre-tittered concentrations. PBS is added to samples before acquisition on a CytoFLEX flow cytometer [22,23]. Additionally, in this analysis, an antibody specific for a typical exosome antigen (CD81) is used to identify exosomes within the pool of extracellular vesicles, and an antibody for PSA is used for the detection of plasmatic exosomes expressing PSA.

The cytometer is calibrated using a mixture of nonfluorescent silica beads and fluorescent (green) latex beads, with sizes ranging from 110 nm to 1300 nm. This calibration step enables the determination of the sensitivity and resolution of the flow cytometer (fluorescent latex beads) and the size of the extracellular vesicles (silica beads). All samples are acquired at low flow rate for the same amount of time in order to obtain an estimate of absolute counts of exosomes that are then comparable between various samples.

## 4. PSA-Expressing Exosomes in PC

In recent years, a critical concept in the field of oncological diseases, liquid biopsy, has emerged. Growing interest in this approach is due to its potential to address some of the problems associated with the acquisition of a histological diagnosis, such as the invasiveness of the biopsy procedure, and the risk of missing pathological tissue.

In PC cases, exosome analysis represents an ideal model for liquid biopsy due to its ability to provide valuable information that may overcome some limitations of the commonly used biomarkers.

In two consecutive experiences, Logozzi et al. demonstrated that analyzing PSA-expressing exosomes provides an incredibly accurate way to discriminate between healthy patients and those with prostate disease, and even within the latter group, it can differentiate between patients affected by BPH and PC [22,23]. The study enrolled a total of 240 patients, divided into three groups of 80 patients (control, BPH, and PC). For each patient, exosomes were extracted from an EDTA-treated blood sample using centrifugation, and their characterization and quantification were performed using a multiple techniques approach.

**Figure 1:**
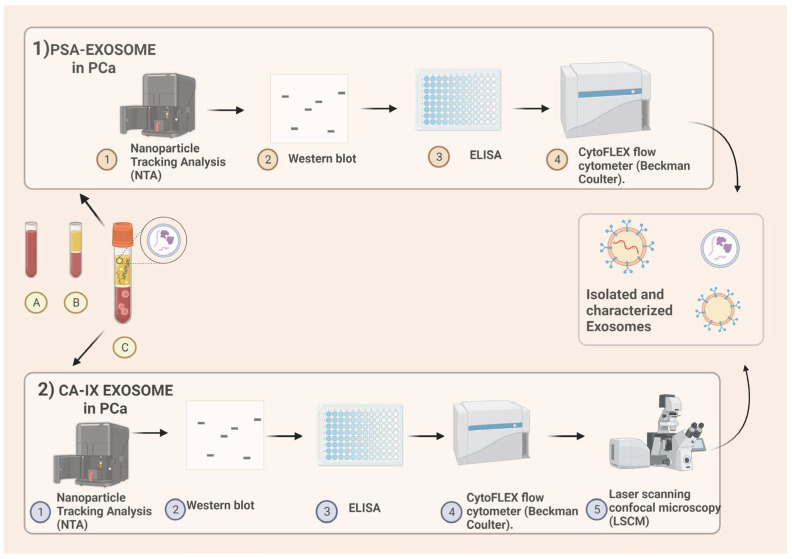
Exosome isolation and characterization in prostate cancer cases. (**1**) PSA exosome extraction. From blood samples, after centrifugation, plasma is obtained. The protocol includes nanoparticle tracking analysis (NTA) for the quality control of plasmatic samples after ultracentrifugation; afterwards, both nanoscale flow cytometry and an immunocapture-based ELISA are used for the extracellular vesicles’ characterization and quantification. In both the analyses, an antibody specific for a typical exosome antigen (CD81) is exploited to identify exosomes within the pool of extracellular vesicles, and an antibody for PSA is used for the detection of plasmatic exosomes expressing PSA. (**2**) Carbonic anhydrase IX exosome extraction. Human plasma samples are collected from EDTA-treated whole blood. Nanoparticle tracking analysis (NTA) is used for size distribution and concentration measurements of exosome samples in liquid suspension. A Western blot analysis is performed. An ELISA for CA is performed, and then the exosomal pH is evaluated using nanoscale flow cytometry. Intracellular acidity is analyzed via confocal microscopy using fluorescent tracers. Figure created with BioRender.com, accessed on 10 May 2023.

The size distribution and concentration of extracellular vesicles in liquid suspension were measured using a nanoparticle tracking analysis (NTA) from Malvern. Western blotting was performed using anti-Tsg101 and anti-CD81 monoclonal antibodies. An ELISA for PSA was carried out using rabbit polyclonal anti-CD81 and mouse anti-PSA HRP-conjugated antibodies. Flow cytometry analysis of exosomes was performed using anti-human CD81 allophycocyanin-conjugated and anti-human PSA fluorescein-conjugated or anti-IgG2a APC and IgG1 FITC antibodies (Figure 1). 

The level of specific PSA exosomes found was able to discriminate between PC patients and non-PC patients (BPH and healthy controls), thereby outperforming the conventional serum PSA test. Specifically, the IC-ELISA alone method achieved a 98.57% sensitivity and a 80.28% specificity in discriminating PC from BPH. The combination of IC-ELISA and NFSC led to an increase of up to 96% in sensitivity and 100% in specificity [23].

Moreover, in the same populations, non-specific exosomes were characterized either in terms of number or size distribution by NTA, and we showed a significant difference between controls and PC patients’ exosome plasma samples for both the non-specific concentration and the size parameters (*p* < 0.0001). We detected a significant increase in the number of exosomes in PC, as well as the shrinking in their size. The standard deviation (SD) of the size distributions was substantially identical in the two population, suggesting a general rigid shift of distribution from controls to PCs. In general, PC exosomes were not only more numerous, but also smaller than the control cases’ exosomes [25].

The ROC analysis performed on the combination of the number and size of plasmatic exosomes showed a maximal sensitivity (89%) and specificity (71%) at cut-off of −0.544. This method allows us to significantly discriminate (AUC = 0.86, *p* < 0.0001) PC patients from healthy cases. At the end, we analyzed the correlation between non-specific and specific exosome-based markers. The specific use based on PSA-expressing exosomes (NSFC-exo) had a statistically significant (but relatively weak) correlation with exosome number, suggesting that the kind of ‘cancer-related’ information provided by both the size and number of exosomes is broadly independent of the specific (prostate) cancer type. Although the “non-specific” (no consideration of PSA expression) predictivity is lower than the specific predictivity of the PSA exosome, it allows for a very considerable predictive power [25].

In the context of PC, miRNa-exosomes have been studied extensively, as they could play a critical role in the development and even progression of the disease. Different cancer-specific miRNa exosomes have been identified in exosomes obtained from biological fluid samples of PC cases [26].

Wang et al. provided a comprehensive review of the utility of exosomal miRNA analysis [26]. They highlighted promising results from blood and urine samples considering miRNAs such as Mir141, Mir-375, and Mir-21, but also Mir-200, Mir574, Mir196A-5P, Mir-501-3p, Mir-2909, Mir-19, Mir-1246, and Lit-7 as potential biomarkers. However, some studies included in this review have yielded inconsistent results, possibly due to a lack of standardization in the exosomal miRNA analysis procedure [27].

Prostate-specific membrane antigen (PSMA) is a zinc metalloprotein encoded by the folate hydrolase 1 gene (FOLH1) and predominantly expressed on the surface of prostate cells. 

In a study conducted by Wang et al., exosome PSMA derived from urine samples of 247 patients (194 individuals with BPH, and 80 with PC) was analyzed. Exosomes were isolated via centrifugation, and subsequently subjected to an enzyme-linked immunosorbent assay (ELISA) using a human glutamate carboxypeptidase 2 (FOLH1)/PSMA ELISA Kit. The analysis of urinary PSMA exosomes revealed a higher diagnostic potential in differentiating PC from BPH, when compared to serum PSA [28].

## 5. Exosomes and Carbonic Anhydrase Expression in PC

Carbonic anhydrase (CA) IX is a zinc-metalloenzyme included in the family of α-carbonic anhydrases (αCA) that catalyze the reversible hydration of carbon dioxide to bicarbonate ions and protons [29]. To date, sixteen human isoforms of αCA have been isolated: five of them have been discovered in cellular cytosol (CA I, II, III, VII, XIII), five are “membrane-related proteins” (CA IV, IX, XII, XIV and XV), two belong to the mitochondria (CA VA and VB), one is secreted in milk and saliva (CA VI), and at least three non-catalytic isoforms have been classified as CA-related proteins (CARP VIII, X and XI). These catalytic isoforms carry out many biological functions, including pH regulation and ion transport in many organs [29]. Of all the isoforms, CA IX is almost uniquely expressed in solid tumors; its presence in non-neoplastic tissues is appreciable exclusively in the gastro-intestinal tract, where it is part of the cell proliferation and differentiation pathways. Cancers are highly active tissues that often contain hypoxic regions and produce large quantities of metabolic acids, so the overexpression of CA IX in solid tumors is regulated by the hypoxia-inducible factor (HIF)-1 [30]. Through the constant release of acid molecules, tumor cells create a hostile environment, which on the one hand favors tumor growth; on the other, it is fatal to the host cells. Increased formation and discharge of protons lead to severe alterations in intracellular and extracellular pH, with important repercussions for tumor growth and progression. This swap in the pH gradient has been shown to occur at an early stage in the malignant transformation pathway [31]. Extracellular pH acidity seems to support tumor progression through alteration of the “pH-dependent modulation” of adhesion between cell and matrix, degrading the matrix itself through the activation of cathepsins and metalloproteases [30]. Moreover, an acidic pH has been shown to suppress immunity function, either via a local strong inhibition of chemotaxis or the prevention of T-cell activation [31]. It has been widely demonstrated through in vitro studies that the cell culture lines exposed to a lower-pH microenvironment (6.5) leads to a greater release of exosomes, compared to the same cells cultured at physiological pH (7.4), independent of the tumor histotype [30,31].

In the last 10 years, there has been an exponential increase in in vitro studies regarding the possible use of CA IX in multiple tumors. Kengo Horie et al. suggested that CA IX contained in exosomes from cell cultures of human renal cell carcinoma (RCC-Caki-1 (JCRB0801), KMRC-, OSRC-2 and 786-O cells) and analyzed using Western blotting and angiogenesis assays promotes cell migration and tube formation [32]. PC shares different biochemical features with other solid tumors (hypoxia, acidity, low nutrient supply, and a low-pH microenvironment) all related to CA IX expression. Therefore, the use of this metalloenzyme as a biomarker or target of possible new therapies represents a concrete objective. In a preliminary study of Logozzi et al. [33], men with histologically confirmed PC and healthy donors (CTR) were compared. The authors aimed to evaluate the level of CA IX expression in exosomes purified from PC and controls. Exosomes were isolated from plasma samples of PC patients and CTR using ultracentrifugation, and characterized through a nanoparticle tracking analysis (NTA) (Figure 1). A subsequent immunocapture-based ELISA assay was performed to quantify and characterize CA IX expression in exosomes. PC patients showed higher plasmatic levels of exosomes than the controls (*p* < 0.1), and exosomes from PC were more homogeneous in size distribution when compared to plasmatic exosomes from controls. CA IX expression was up-regulated in exosomal purification lysates from PC plasma when compared to the exosomal fractions of control plasma. Moreover, authors showed that the CA activity/mg protein found in exosomes isolated from PC plasma (2.9 ± 0.4) was 2.4-fold higher when compared to exosomes purified from CTR plasma (1.2 ± 0.2) (*p* < 0.0001). The results revealed that CA IX-positive exosomes were 25-fold higher in plasma samples from PC patients (558 ± 90) than in those from CTR (22 ± 2), (*p* < 0.0001) [33]. This study aimed to demonstrate not only the increased levels of CA IX in exosomes of PC patients, but also the direct correlation between the acidic microenvironment, the release of exosomal particles in the extracellular compartment, and the upregulation of CA IX expression and activity.

## 6. Conclusions

The main limitation to the current use of PSA as a routine marker for the early diagnosis of PC is its lack of specificity in distinguishing between PC and BPH. The organ specificity and not disease specificity of serum PSA reduces the possibility of differentiating between two diseases, such as PC and BPH, that often coexist in the same patient. In our clinical practice, we very often evaluate patients who have significant BPH-related PSA elevations in the absence of a PC. On this point, the use of exosome detection can improve the specificity of PSA as a marker for the early diagnosis of PC. The analysis of PC-linked EV through liquid biopsy represents a promising diagnostic model that can effectively distinguish patients with PC from those with non-malignant prostatic disease or healthy individuals. Various biomolecules characteristic of different exosomes have emerged as potential biomarkers for PC, sustaining their potential use in clinical practice. Notably, the analysis of PSA exosomes obtained from blood samples has demonstrated remarkable sensitivity and specificity when compared to traditional serum PSA analysis. Quantitative and qualitative analysis, defined as the search for EV expressing specific antigens, represents a promising frontier in the search for new and reliable diagnostic and disease progression biomarkers that could contribute to innovation in current clinical practice. In this context, there are several basic research studies on the role of exosome-associated proteins and their functional relevance in PCa invasion and metastasis [34]. Although these studies have been conducted on synthetic cell lines, their results are very encouraging, and in the near future, these markers may be used in clinical practice. We also suggest more simply that plasmatic exosomes from PC cases are more numerous and smaller in size compared to those in healthy subjects. To improve the specificity of PSA exosomes, prostatic fluid has been proposed as a sample from which to obtain these exosomes. The levels of some miRNA in semen were found to be higher in prostate cancer patients when compared with normal healthy individuals [35]. Prostatic fluid appears to have potential use in clinical practice; however, to date, only a few studies have analyzed the expression level of miRNAs in semen.

NTA analysis performed on a combination of the size and number of plasmatic exosomes can significantly distinguish between PC and BPH cases. However, to date, only a few studies have been conducted on isolated cohorts of patients. The use of exosome analysis in clinical practice is currently limited by several issues, including a lack of standardization in the analytical process, and high costs (which are still too high for large-scale use). In the presence of absolutely promising results, the real obstacle for the diffusion of this analysis in clinical practice is a methodology that requires high costs and prolonged times. The characteristics of exosomes make analytical simplification difficult. It is reasonable to anticipate that future research will continue to explore this new area of medicine, leading to the development of new methodologies that will enable liquid biopsy through exosome analysis to become a routine diagnostic tool in PC diagnosis.

## Figures and Tables

**Table 1:** Assays for exosome characterization and quantification in patients with prostate cancer.

Assay	Minimum Detectable Vesicle Size (nm)	Advantages	Limits	Reference
Nanoparticle Tracking Analysis (NTA)	70–90	- Precise measurement of the concentration and size of the particles - Highly reliable and sensitive method - More accurate size information - Information on biological protein biomarkers related to exosomes	- Resolution of multimodal samples is relatively limited - Risk of overestimating particle concentration - Proper dilution of the sample for measurement purposes	[22,23]
Resistive pulse sensors (RPS)	70–100	- Quick and easy to perform	- Accurate sizing of vesicles by RPS requires a low and negligible electrical conductivity of a particle - The major concerns with RPS are pore clogging and pore stability	[18,19,20,21,24]
Conventional flow cytometry	270–600	- Knowledge of the analyzed sample volume and the particle concentration can be determined without calibration with beads - Easy and fast to perform	- Accuracy limited by the refractive index of the vesicles	[20,21,22,23]
Dedicated flow cytometry	150–190	Relatively fast, and allows multiplex fluorescence detection	Less accurate on vesicles, owing to heterogeneity of the refractive index of vesicles	[20,21,22,23]
Transmission electron microscopy (TEM)	1	- High resolution	- Measurement conditions and sample preparation - Extensive and multi-step preparation needed	[24,20]

## Data Availability

Not applicable.

## Abbreviations

PC	prostate cancer
EV	extracellular nanovesicles
BPH	benign prostatic hyperplasia
CA IX	carbonic anhydrase nine
DRE	digital rectal examination
PSA	prostate-specific antigen
AUC	area under the curve
TMPRSS2	fusion trans-membrane serine protease 2
FISH	fluorescence in situ hybridization
NTA	nanoparticle tracking analysis
PSMA	prostate-specific membrane antigen
FOLH1	folate hydrolase 1 gene
FFPE	formalin-fixed paraffin-embedded
RT-PCR	reverse-transcriptase polymerase chain reaction
